# Supplementary material for: Improving the Safety of N,N-Dimethylacetamide (DMA) as a Potential Treatment for Preterm Birth in a Pregnant Mouse Model Using a Vaginal Nanoformulation
Source: bioRxiv. 2025 Jan 20:2025.01.16.633348. Preprint. [Version 1] doi: 10.1101/2025.01.16.633348 (PMC11785104; doi:10.1101/2025.01.16.633348)
Supplement: Supplement 1 — Supplemental Figure 1: Semi-quantitative Histology Workflow. Sections were analyzed with a Nikon Eclipse Ts2R microscope and micrographs were captured with a Nikon Digital Sight 10 camera. Digitized images (400x magnification) were loaded into ImageJ/Fiji version 2.14.0 and underwent image deconvolution where they were separated into their respective channels and background subtraction was done by subtracting out pixels that did not fall within a range of color/pixel “intensities” to generate a binary image where the area of the pixels of interest can be measured. The “H&E Color Deconvolution Macro” was used for red blood cell area count as depicted in panels A-C. The “H-DAB Color Deconvolution Macro” was used for CD31 area count as depicted in panels D-F. Supplemental Figure 2: Sexual Dimorphism Does Not Play A Role in Placental Abnormalities Following DMA Administration. Analysis within treatment groups revealed no significant difference in the severity of labyrinth congestion between placentas attached to female pups and placentas attached to male pups [Panels A-D]. Similarly, no significant difference was observed when comparing CD31 loss in placentas attached to female pups versus placentas attached to male pups [Panels E-H] (n=12–28/treatment group) [file media-1.docx]

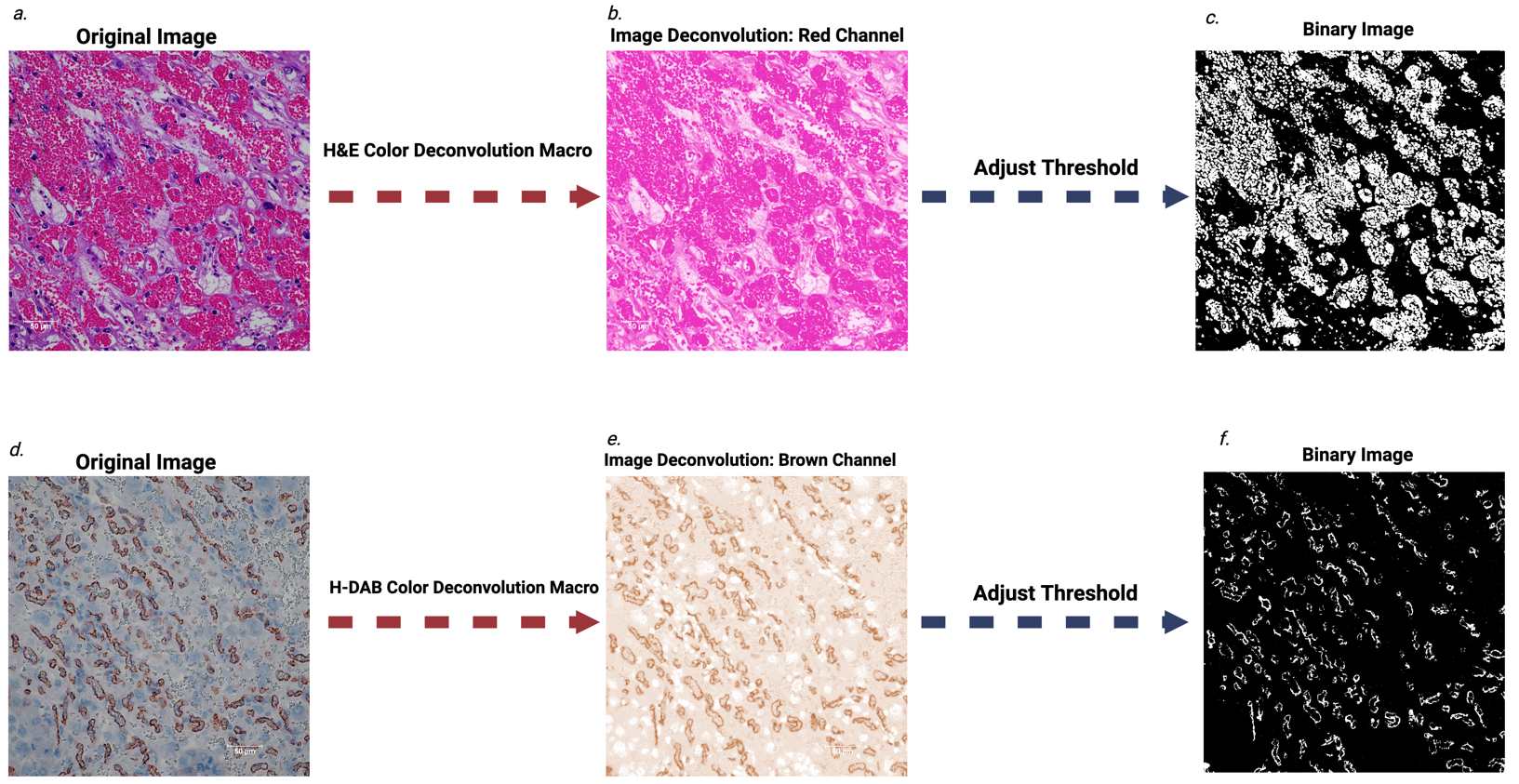


**Supplemental Figure 1: *Semi-quantitative histology workflow.*** *Sections were analyzed with a Nikon Eclipse Ts2R microscope and micrographs were captured with a Nikon Digital Sight 10 camera. Digitized images (400x magnification) were loaded into ImageJ/Fiji version 2.14.0 and underwent image deconvolution where they were separated into their respective channels and background subtraction was done by subtracting out pixels that did not fall within a range of color/pixel “intensities” to generate a binary image where the area of the pixels of interest can be measured. The “H&E Color Deconvolution Macro” was used for red blood cell area count as depicted in* ***panels A-C****. The “H-DAB Color Deconvolution Macro” was used for CD31 area count as depicted in* ***panels D-F.***


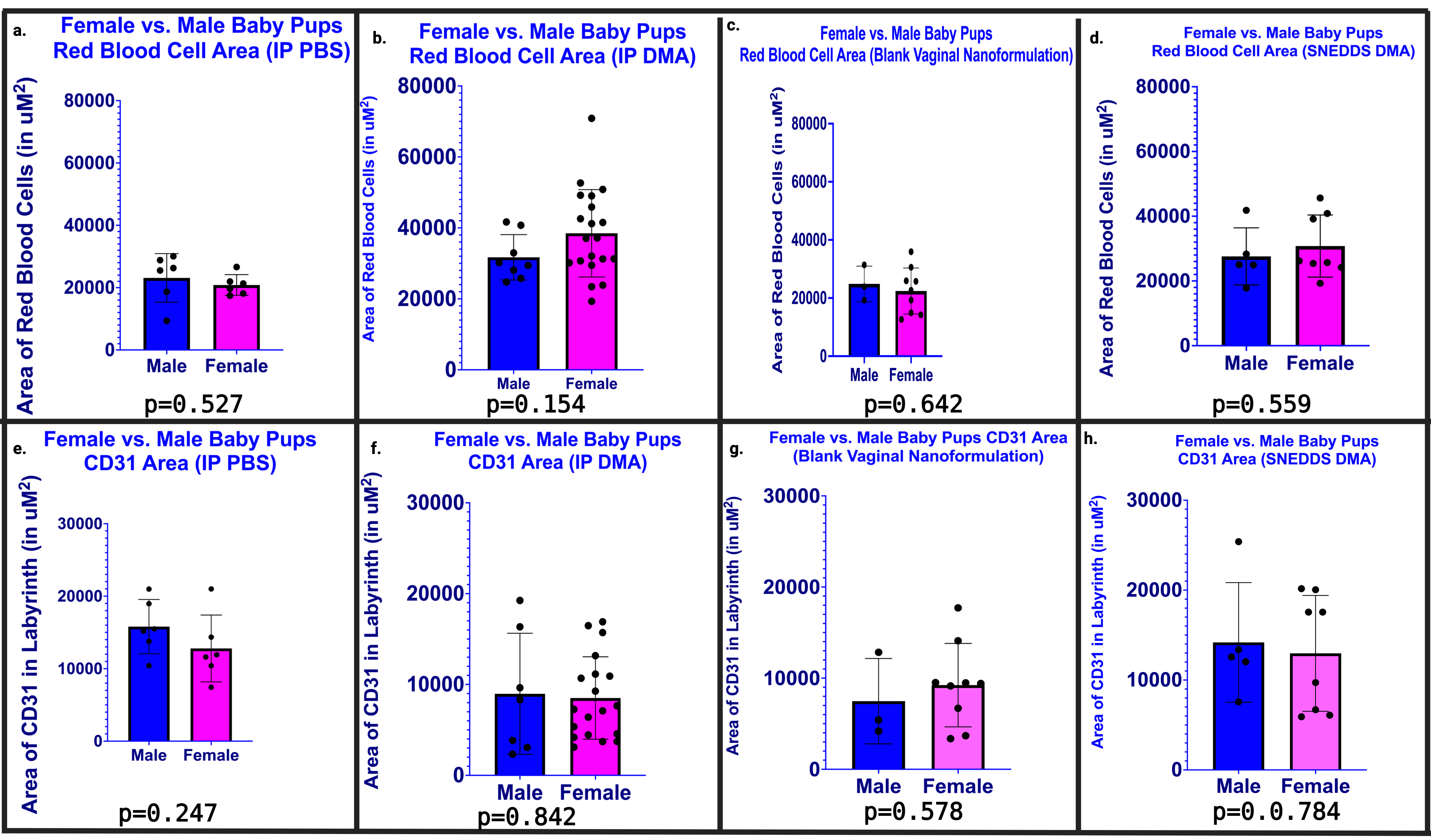


**Supplemental Figure 2: Sexual dimorphism does not play a role in DMA’s effect on placental histomorphology.** *Red blood cell areas were calculated in placental sections from each of the four groups, as indicated, and results for male and female palcentas were compared*  [***Panels A-D].*** *Similarly, CD31staining areas were calculated in placental sections from each of the four groups, as indicated, and results for male and female palcentas were compared* ***[Panels E-H].*** *(N=12-28/treatment group.)*
